# Cold‐seeking behaviour mitigates reproductive losses from fungal infection in *Drosophila*

**DOI:** 10.1111/1365-2656.12438

**Published:** 2015-10-16

**Authors:** Vicky L. Hunt, Weihao Zhong, Colin D. McClure, David T. Mlynski, Elizabeth M.L. Duxbury, A. Keith Charnley, Nicholas K. Priest

**Affiliations:** ^1^ Milner Centre for Evolution and Department of Biology and BiochemistryUniversity of BathClaverton DownBathBA2 7AYUK

**Keywords:** behavioural anapyrexia, behavioural fever, *Drosophila melanogaster*, Fecundity, life‐history traits, *Metarhizium robertsii*, temperature preference

## Abstract

1. Animals must tailor their life‐history strategies to suit the prevailing conditions and respond to hazards in the environment. Animals with lethal infections are faced with a difficult choice: to allocate more resources to reproduction and suffer higher mortality or to reduce reproduction with the expectation of enhanced immunity and late‐age reproduction. However, the strategies employed to mediate shifts in life‐history traits are largely unknown.
2. Here, we investigate the temperature preference of the fruit fly, *Drosophila melanogaster*, during infection with the fungal pathogen, *Metarhizium robertsii*, and the consequence of temperature preference on life‐history traits.
3. We have measured the temperature preference of fruit flies under different pathogen conditions. We conducted multiple fitness assays of the host and the pathogen under different thermal conditions. From these data, we estimated standard measures of fitness and used age‐specific methodologies to test for the fitness trade‐offs that are thought to underlie differences in life‐history strategy.
4. We found that fungus‐infected fruit flies seek out cooler temperatures, which facilitates an adaptive shift in their life‐history strategy. The colder temperatures preferred by infected animals were detrimental to the pathogen because it increased resistance to infection. But, it did not provide net benefits that were specific to infected animals, as cooler temperatures increased lifetime reproductive success and survival whether or not the animals were infected. Instead, we find that cold‐seeking benefits infected animals by increasing their late‐age reproductive output, at a cost to their early‐age reproductive output. In contrast, naive control flies prefer warmer temperatures that optimize early‐age reproductive, at a cost to reproductive output at late ages.
5. These findings show that infected animals exhibit fundamentally different reproductive strategies than their healthy counterparts. Temperature preference can facilitate shifts in strategy, but not without inevitable trade‐offs.

## Introduction

Infections can impose severe costs on the host. Parasites not only directly compete for host resources and trigger energetically costly host immune responses, but can also cause dramatic reductions in host reproductive output. Infected hosts have been shown to both increase and decrease their reproductive outputs (Hurd 2001; Velando, Drummond & Torres 2006). However, such reproductive changes as a result of infection are hard to interpret as they could represent the inevitable by‐products of infection (costs of parasitism), active manipulation of host resources by parasites (Webb & Hurd 1999; Hurd 2003) or adaptive shifts in life‐history strategy of the host (Forbes 1993; Hurd 2001). Thus, it is not clear how animals mitigate the reproductive consequences of infection.

Hosts can respond to the threats of parasites in a myriad of ways beyond their immune system as traditionally defined. Although often overlooked in studies of immunity, ‘non‐immunological defences’ including behavioural mechanisms of defence can be effective mechanisms of immunity (Thomas & Blanford 2003; Parker *et al*. 2011; De Roode & Lefèvre 2012). Furthermore, behaviours may be employed to combat the negative effects of an infection by, for example, altering life‐history strategy to optimize reproductive success. However, the adaptive significance of non‐immunological immunity has been difficult to assess. Practically, it is often difficult to experimentally manipulate behaviour, in contrast to the molecular and genetic tools available for components of the innate immune system. Host reproductive changes as a result of infection are also hard to interpret as they could represent the costs of mounting immune responses (Sheldon & Verhulst 1996; Schmid‐Hempel 2003), shifts in life‐history strategy towards fecundity compensation or reduction (Forbes 1993; Hurd 2001), and even active manipulation of host resources by parasites (Webb & Hurd 1999; Hurd 2003).

Previous studies of thermoregulatory behaviour have found that infected poikilotherms exhibit warm‐seeking (behavioural fever (Watson 1993; Adamo 1998; Elliot, Blanford & Thomas 2002; Hunt & Charnley 2011; Hunt *et al*. 2011), cold‐seeking (behavioural anapyrexia (Müller & Schmid‐Hempel 1993; Zbikowska & Cichy 2012)) and sometimes both behaviours (Anderson, Blanford & Thomas 2013). However, these studies have largely focussed on the survival and immune function of infected hosts paying little attention to host reproductive fitness in general, particularly the comparative fitness of non‐infected control animals. If the response to infection represents an adaptive shift in life‐history strategy, we would expect that infected animals would benefit appreciably more from inhabiting the preferred temperature than control animals; we would expect that the temperature preference would provide benefits one life‐history trait at a cost to other traits; and we would expect that the response ultimately results in increased offspring production for infected animals. This is especially of interest to ecological immunologists as all host defence traits are expected to be costly and trade‐off with other host life‐history traits (Sheldon & Verhulst 1996; Schmid‐Hempel 2003).

Here, we report the temperature preference of the fruit fly, *Drosophila melanogaster*, under control conditions and when exposed to the entomopathogenic fungus, *Metarhizium robertsii*. We then examine the reproductive and survival consequences for the hosts in a fully factorial design in which we manipulate the thermal regimes to mimic the temperatures preferred by infected and control animals in combination with infection treatments. This system is ideal for this problem because: (i) *D. melanogaster* is a model species for the study of life history (Prasad & Joshi 2003), temperature preference (Dillon *et al*. 2009), innate immunity (Lemaitre & Hoffmann 2007), resistance, the ability to reduce pathogen load, and tolerance, the ability to limit the impact of infection (Ayres & Schneider 2009), and the interactions between temperature and immunity (Lazzaro *et al*. 2008; Linder, Owers & Promislow 2008); (ii) *M. robertsii* is a common insect pathogen that has previously been shown to induce behavioural fever in locusts (Elliot, Blanford & Thomas 2002; Ouedraogo 2003) and drives reproductive and survival costs in fruit flies (Zhong *et al*. 2013; McClure *et al*. 2014).

## Materials and methods

### Culture Maintenance and Infection Treatment

All experiments used the Oregon‐R strain that had been cultured at 25 °C, 40% RH and 12 : 12 light/dark cycle on standard Nipagin‐infused (an antifungal agent) oatmeal‐molasses‐agar media supplemented with a single grain of live baker's yeast. We cultured *M. robertsii* (isolate ARSEF 2575, previously classified as *M. anisopliae* strain ME1) at 28 °C in continuous light on one quarter strength Sabouraud dextrose agar with additional yeast extract (SDAy) and collected spores after 7–14 days. Flies were topically infected by gently shaking cohorts of 10–15 flies in a 250‐mL conical flask containing 900 μg of live spores for 10 s*. Metarhizium*‐infected flies were sequentially transferred to fresh vials containing standard media to minimize the transfer of excess spores to the experimental vials. Control and heat‐killed pathogen treatments were handled identically, except that the flask was empty or contained 900 μg of autoclaved (121 °C, 15 min) spores, respectively.

### Temperature Preference of Drosophila

Temperature preference of flies was measured on a purpose‐built apparatus (Fig. S1a, modified from Sayeed & Benzer 1996). Four escape‐proof experimental lanes were created along the length of the apparatus with a perspex lid, and the application of the insect deterrent Fluon^®^ to the inner walls encouraged flies to stay on the surface of the aluminium block. A piece of white paper placed on the aluminium block and marked into 10 equal sections was used to identify the position of flies across the gradient. A k‐type thermocouple (Omega Engineering) was used to measure the mid‐point of each section across the apparatus, which confirmed a linear temperature gradient ranging from 16 to 32 °C along the aluminium block (Fig. S1b).

We transferred control or infected mixed sex flies without anaesthesia in groups of 10–15 flies into the apparatus at 23·5, 47·5 and 71·5 h post‐infection. Temperature preference of each fly was established by recording their locations along the temperature gradient after 30 min. All flies were measured only once, and the apparatus was cleaned with 70% ethanol after each trial, and the paper marking the 10 sections was replaced. Temperature preferences were always assessed between 11 : 00 and 14 : 00 with a minimum of four trials for each pathogen treatment/time point. We also assessed the distribution of flies in the absence of thermal gradient at room temperature (Fig. S1c).

### Effect of Temperature on Host Survival and Age‐Specific Mortality

We treated 3‐ to 4‐day‐old mixed sex adult flies with live, heat‐killed or control pathogen treatment. Flies were then maintained in population cages (11 cm diameter × 15 cm height) at 22 or 25 °C until all animals had died (*n* = 50 cages with approximately 40 flies/cage). The cages were provided with fresh fly media vials daily, and dead flies were removed and recorded. Cause of death was confirmed by random sampling of cadavers and examining them for signs of *Metarhizium*‐like fungal growth. Sampled cadavers were surface sterilized (brief sequential immersion in 1% bleach, 70% ethanol and sterile water) and placed on filter paper moistened with sterile water in sealed Petri dishes. The resulting plates were kept at 28 °C for up to 10 days to check for external fungal growth under a dissection microscope.

### Life‐history Consequence of Temperature Preference and Infection

Two‐day‐old adult virgin females were allowed to mate with males for 24 h at 25 °C in groups of 20 flies. Males were then discarded, and females were maintained for a further 24 h at 25 °C. We treated females (adults, age 4 days; *n* = 259) with one of three pathogen treatment (control, live and heat‐killed fungus) and transferred them into individual vials containing fresh media in 10 randomized blocks at 22 or 25 °C. Every 2 days post‐infection, we counted the number of eggs laid by each female and replaced the food media with fresh vials until the fly had died. The number of adult deaths was also recorded after each egg collection. We incubated vials containing eggs at 25 °C and examined them again after 14 days to record the number of eclosed pupae.

### Effects of Temperature on Fungal Growth, Host Resistance and Tolerance

We assessed the growth rates of *M. robertsii* both on artificial media and live hosts. For *in vitro* growth, we placed 4 mm non‐sporulating mycelial plugs in the centre of SDAy plates at 22, 25 and 28 °C in continuous darkness (*n* = 30). The diameter of fungal mycelium was measured daily along two perpendicular axes drawn on the Petri dish, for a total of 8 days. We estimated the *in vivo* growth rate of *M. robertsii* from periodic sampling of infected host pathogen load. Mixed sex fruit flies were first exposed to 20–25 mg of *M. robertsii* according to the infection protocol and maintained in population cages (approximately 350 flies/cage) that were designed to allow easy access and sampling of flies with a pooter. Treated flies were then placed at five temperatures ranging from 18 to 28 °C over a period of 17 days (*n* = 6 cages per treatment). We randomly sampled pairs of surviving flies on day 3, 5 (28 °C treatment only), 7, 10, 14 and 17 post‐infection for all temperature treatments (*n* = 147 flies). Sampled flies were individually surface‐sterilized (brief immersion in 1% bleach, 70% ethanol and sterile water) and homogenized in a buffer of 0·04% Tween^®^80. The homogenates were spread on fresh SDAy plates and incubated at 28 °C for 3 days after which *M. robertsii*‐like colony‐forming units (CFUs) were identified by spore morphology using a microscope and counted. In cases where the number of CFUs on the plate was too large to count (>700), pathogen load was estimated by multiplying the mean CFUs within 1 cm^2^ sample squares by the number of squares. For host mortality rates, we recorded the daily number of deaths from the same fly populations that we used to sample flies for estimating pathogen load.

### Statistical Analysis

All statistical analyses were carried out with spss version 13.0 and r version 2.11.1 (R Core Team 2012) (see Supporting Information, Appendix S1 for further details on statistical analyses).

## Results

### Temperature Preference of Drosophila

**Figure 1:**
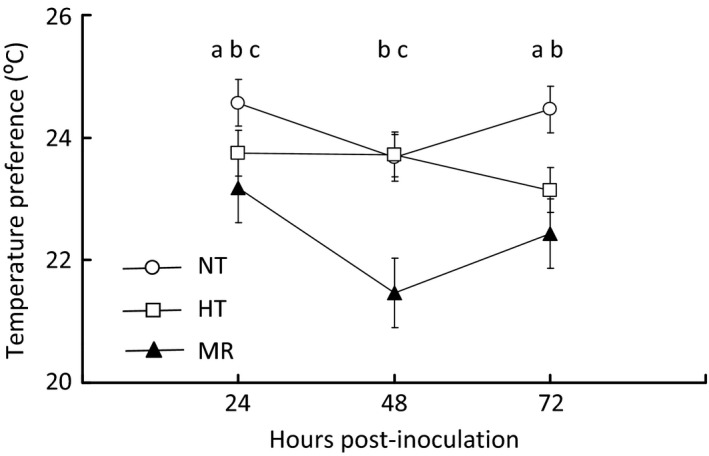
*Drosophila melanogaster* infected with *Metarhizium robertsii* prefer colder temperatures relative to uninfected control animals. Flies were topically exposed to live *M. robertsii* spores (MR, triangles), heat‐killed spores (HT, squares) or sham controls (NT, circles) and placed on a temperature gradient ranging from 16 to 32 °C at 3 time points post‐infection (24, 48 and 72 h). All data represent mean ± SE. Letters indicate significance (*P* < 0·05, *χ* ^2^ test) for pairwise comparisons: (a) NP & HP; (b) HP & LP; (c) NP & LP.

When placed on a linear temperature gradient extending from 16 to 32 °C (Fig. S1a–c), control flies typically preferred ~25 °C (Fig. S2), but switched their preference to colder temperatures (~22 °C) within 24 h after topical exposure to live *M. robertsii* spores. This behavioural anapyrexia persisted until at least 72 h post‐infection (Live pathogen vs. no pathogen control: *χ* ^2^ > 15·2, *P* < 0·002 at all time points). Interestingly, flies treated with heat‐killed spores also exhibited behavioural anapyrexia at 24 and 72 h post‐infection, though to a lesser degree than live pathogen‐treated flies (Heat‐killed pathogen vs. No pathogen: 24 h, *χ* ^2^ = 8·22, *P* = 0·04; 72 h, *χ* ^2^ = 12·0, *P* = 0·007) (Fig. 1).

### Effects of Temperature Preference on Host Reproductive Fitness

**Figure 2:**
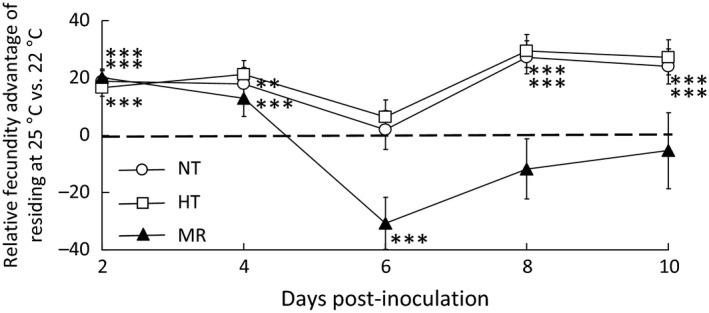
A cooler temperature (22 °C) provides an age‐specific fecundity advantage to infected but not healthy fruit fly. Control flies and flies treated with heat‐killed spores have higher fecundity at 25 than 22 °C for all early‐age intervals. Flies exposed to live pathogen have lower fecundity at 22 °C for the first interval and have similar fecundity over subsequent intervals. The dashed line indicates where fruit flies achieve the same fecundity at 22 and 25 °C. Asterisks indicate a significant difference between 22 and 25 °C at that time point: **, *P* < 0·01; ***, *P* < 0·001 (Welch two sample *t*‐test).

Pathogen‐induced behavioural anapyrexia resulted in an initial reduction, followed by an increase in host reproductive output. In particular, temperature interacted with infection status to influence the age‐specific pattern of the production of eggs (Age × Temperature × Pathogen: *F*_2,4923_ = 15·5, *P* < 0·0001; Fig. 2; Table S1). From a peak at 2–4 days post‐infection, egg production declined rapidly with age in all treatments (*F*_1,4923_ = 218·3, *P* < 0·0001). Flies residing at 25 °C had significantly higher early‐age fecundity at the expense of much lower late‐age fecundity than those at 22 °C (Age × Temperature, *F*_1,4923_ = 77·3, *P* < 0·0001). However, while the fecundity patterns of infected flies were initially similar to the control treatments at the two temperatures, as the infection progressed (after 4–6 days post‐infection), the decline in fecundity was much slower for infected animals residing at 22 °C than those at 25 °C (Live pathogen: Age × Temperature, *F*_1,237_ = 9·8, *P* = 0·002; Fig. S3c; Table S2). Thus, infected flies residing at their preferred temperature initially exhibited reduced reproductive output, compared to infected flies at the warmer temperature, but achieved substantially greater reproductive output at later‐ages (Fig. 2).

In contrast, anapyrexia does not stimulate a rapid propagation strategy, as flies residing at 22 °C always achieved lower intrinsic rates of increase (*r*) than flies at 25 °C regardless of infection status (Temperature, *F*_1,24_ = 19·4, *P* < 0·001; Pathogen × Temperature, *F*_2,24_ = 1·00, *P* = 0·384; Fig. S4a; Table S3). There was also no concrete evidence that anapyrexia increases total reproductive output. Infected flies residing at 22 °C achieved significantly higher LRS in the live pathogen treatment, but a similar trend was observed in the control treatment. Though there was weak evidence that inhabiting cooler temperatures increases LRS (*F*_1,227_ = 4·0, *P* = 0·047), there was no evidence that infected animals benefited more from inhabiting the cold (Pathogen × Temperature, *F*_2,227_ = 1·87, *P* = 0·157; Tables S4b and S3).

### Effect of Temperature on Host Survival and Age‐Specific Mortality

**Figure 3:**
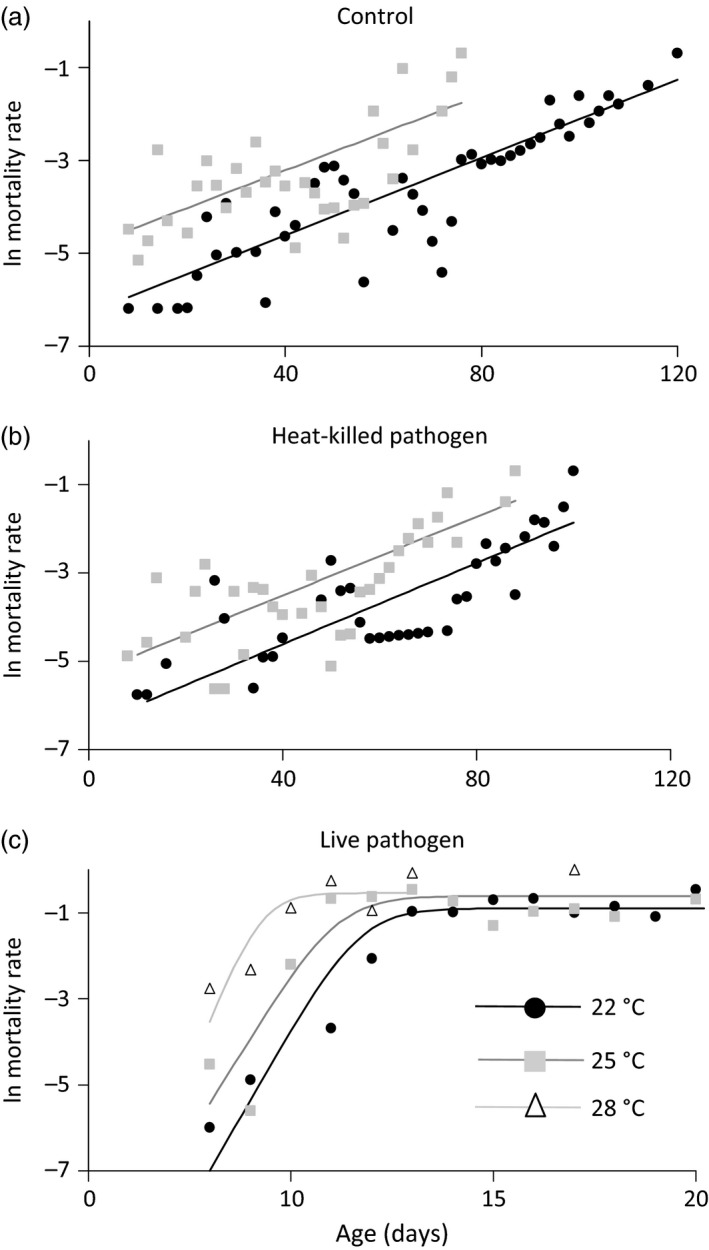
Temperature influences age‐independent mortality while *Metarhizium robertsii* infection affects age‐dependent mortality in Drosophila. (a) Sham controls. (b) Flies exposed to heat‐killed *M. robertsii* spores. (c) Flies exposed to *M. robertsii* spores. Data shown are natural‐log‐transformed daily mortality rates. Fitted lines are Gompertz (a and b) and Logistic mortality models (c).

We found that anapyrexia‐like temperatures (22 °C) extend post‐infection survival times, but the warmer temperature preferred by control flies (25 °C) elevates their risk of death. Independent survival experiments confirmed that exposure to live fungal spores reduces the survival of flies (Pathogen, $\chi_{2}^{2}$ = 925, *P* < 0·0001), and that residing at 22 °C confers survival benefits to infected flies relative to staying at 25 °C (Temperature, $\chi_{1}^{2}$ = 214, *P* < 0·0001; Fig. S5). However, infected flies do not benefit proportionally more from colder temperature than untreated control or heat‐killed fungus treated flies (Pathogen × Temperature, $\chi_{2}^{2}$ = 3·11, *P* = 0·21). The same pattern is confirmed by age‐specific analysis of mortality rates. In contrast to the control and heat‐killed pathogen treatments, which fit simple Gompertz mortality trajectories (Fig. 3a,b), exposure to live pathogens significantly increases the age‐dependent mortality rate and causes a prolonged period of mortality ‘levelling off’, justifying more complex logistic mortality models ($\chi_{1}^{2}$ > 2·7, *P* < 0·05 for live pathogen treatment at all temperatures; Fig. 3c). However, the midlife flattening of age‐specific mortality rate we observe for pathogen‐treated flies is different to the late life‐plateaus observed for controls, which showed more conventional patterns of ageing. Colder temperature is only associated with reduced background (age‐independent) mortality, and this effect was found in all pathogen treatments ($\chi_{1}^{2}$ > 7·8, *P* < 0·001 for all treatments).

### Effects of Temperature Preference on Parasite Growth

**Figure 4:**
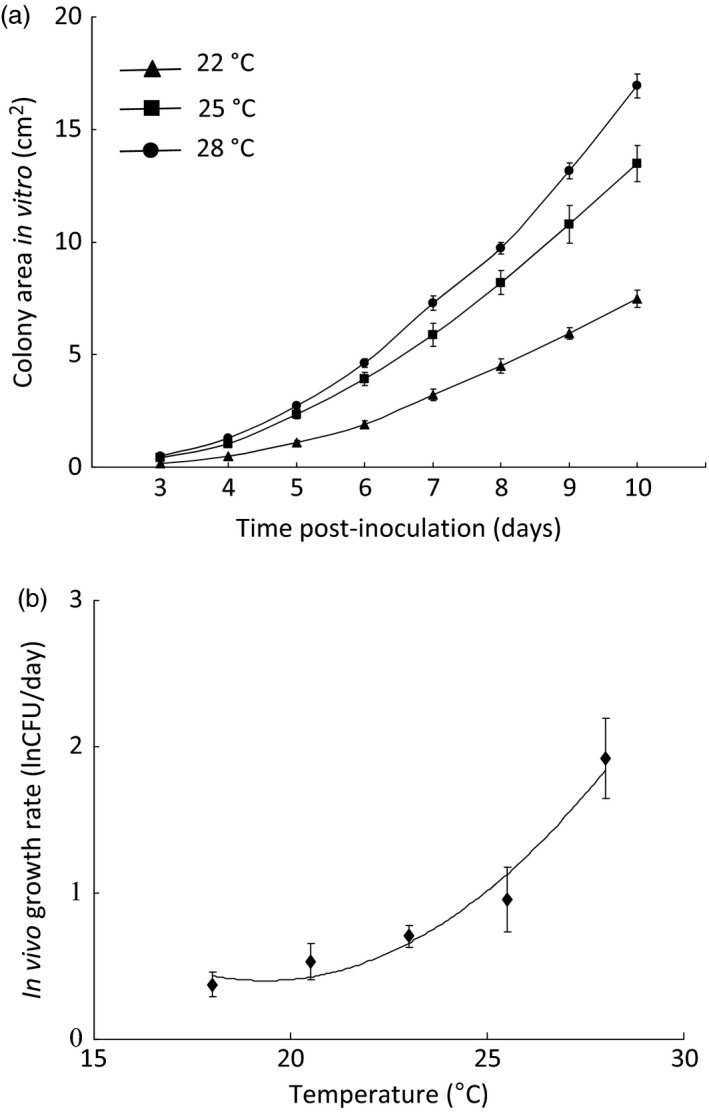
Moving to colder temperatures is detrimental to the fungal pathogen; (a) *in vitro* colony growth of *Metarhizium robertsii* at 22, 25 and 28 °C (*Post hoc* Tukey's HSD tests revealed all pairwise comparisons were significant at *P* < 0·0001); (b) *in vivo* rate of growth of the fungal pathogen at 18, 20·5, 23, 25·5 and 28 °C. Samples of live flies were taken at 3–4 day intervals, and pathogen load was established by counting the number of colony‐forming units (CFUs) on replicate fungal media plates. The line of best fit represents the least squares polynomial regression for natural‐log‐transformed CFU counts. All data represent mean ± SE.

Colder temperatures reduced fungal growth rate both *in vitro* and *in vivo* (*F*_2,15_ = 68·9, *P* < 0·0001; *F*_1,26_ = 21·7, *P* < 0·0001; Fig. 4a,b) and increased the time taken to reach peak pathogen load in the live host (*F*_1,18_ = 48·5, *P* < 0·0001). Thus, suggesting that cold‐seeking behaviour is an effective mechanism of resistance against fungal infections. The rise and subsequent fall in pathogen load occurred more rapidly at warmer temperatures; consequently, the pathogen load at any particular temperature depended greatly on the time post‐infection at which it was measured (Temperature × Age, *F*_4,103_ = 3·40, *P* = 0·012; Table S4. Using mixed effects regression models, we found that while both higher pathogen load (*F*_1,98_ = 4·57, *P* = 0·035) and warmer temperatures (*F*_4,25_=7·7, *P* = 0·026) increased the host's risk of death, moving to the colder temperatures did not enhance tolerance, characterized here as the capacity of the host to mitigate the harmful effects of fungal infection on mortality (Pathogen load × Temperature, *F*_4,98_ = 0·96, *P* = 0·43; Fig. S6, Table S5).

## Discussion

‘Non‐immunological’ mechanisms such as thermoregulatory behaviour are increasingly appreciated as critical components of an animal's defence against pathogens (Thomas & Blanford 2003; Parker *et al*. 2011; De Roode & Lefèvre 2012). However, the adaptive significance of non‐immunological defences has been difficult to test. We find that when exposed to a common insect fungal pathogen, the fruit fly alters its temperature preference, which helps to mitigate the loss of fitness associated with infection.

### A Novel Mode of Immunity in Drosophila

Despite being one of the most important model organisms for the study of temperature preference (Dillon *et al*. 2009) and innate immunity (Lemaitre & Hoffmann 2007), little is known about whether *Drosophila* employ thermoregulatory behaviour to fight infections. In contrast to the well‐documented phenomenon of behavioural fever (Watson 1993; Adamo 1998; Elliot, Blanford & Thomas 2002; Hunt *et al*. 2011), but consistent with other cases of behavioural anapyrexia (Müller & Schmid‐Hempel 1993; Zbikowska & Cichy 2012), we find that *D. melanogaster* infected with the fungus *M. robertsii* preferred colder temperatures compared with uninfected control animals. This switch in temperature preference bestows long‐term fitness benefits for the host, thus implying behavioural anapyrexia is host driven. Though lower temperatures reduced the growth rate of the fungus, we cannot rule out the possibility that this was not also advantageous to pathogen transmission and moving to a colder temperature could, for example, reduce predation of the fly, which could in turn increase the transmission success of the fungus. Further work will be required to investigate such possibilities. To a lesser degree, flies treated with a heat‐killed fungus also preferred colder temperatures. This suggests that the behavioural anapyrexia is host driven, because a dead pathogen would not be able to manipulate its host. It is possible that unknown factors associated with the presence of a dead fungus on the surface of the cuticle could induce a mild behavioural anapyrexia. Confirmation of the activation of other aspects of the immune system, such as antimicrobial peptides, would indicate the host has identified pathogenic material on its surface, thus supporting this behaviour is host driven.

Behavioural anapyrexia by infected fruit flies directly enhances resistance against *Metarhizium* infections by placing the fungus in a suboptimal thermal environment reducing its germination success and vegetative growth (this study, Ouedraogo *et al*. 1997; Tefera & Pringle 2003; Dimbi *et al*. 2004). Given the universal affect of temperature on microbial replication rates, anapyrexia is likely to act as a non‐specific mechanism of resistance that could be effective against numerous pathogens. However, in some cases the thermal response of an insect to infection varies depending on the susceptibility of the pathogen to temperature (Adamo 1998). There is a growing body of evidence to support an increase of immune gene expression at colder temperatures in Drosophila and other invertebrates (Linder, Owers & Promislow 2008; Murdock, Moller‐Jacobs & Thomas 2013; Sinclair *et al*. 2013). Although we did not investigate the possibility in this study, moving to the cold could further augment host immune function through its effect on immune gene expression. To date, many studies investigating thermal relationships of immune gene expression have focused on temperatures below the ~22 °C observed here for behavioural anapyrexia. It is important that future studies also consider temperatures and thermal regimes closer to those realistically experienced by fruit flies to fully understand the interactions between temperature and immune gene expression during an infection.

Previous work suggests that tolerance to biotic and abiotic stress increases at colder temperatures (Sinclair *et al*. 2013). However, we found no evidence that the colder temperature enhances tolerance to fungal infection, which suggests that resistance and tolerance can vary independently in our system (Ayres & Schneider 2008). Our measure of tolerance, defined as age‐specific reaction norms between pathogen load and host mortality risk (Baucom & de Roode 2011), could be biased by cohort heterogeneity in resistance to infection if highly susceptible flies have higher pathogen loads than those that survive, which would make the correlation between observed pathogen load and mortality rate dependent on the severity of the infection. Measurements of fitness loss per individual could offer an alternative to our age‐specific mortality‐based measurements because it allows for individual, rather than cohort, measures of tolerance; however, because it does not account for the sensitivity of reproductive output to temperature and cannot be measured in our system without destructive sampling, it could produce misleading results.

The results we report here are based on the temperature preference of small cohorts of flies. The behaviour of animals, including *D. melanogaster,* can be influenced by collective group behaviour (Berdahl *et al*. 2013; Ramdya *et al*. 2014), and it is therefore important to note that we can only confidently state that the behavioural anapyrexia observed here applies to groups of fruit flies and the result for individual flies may differ, if, for example, an individual was searching for a mate. We hope this study will provide a foundation for future studies on thermal preferences in *D. melanogaster* during infection. To establish the extent of behavioural anapyrexia, further work exploring the incidence of cold‐seeking behaviour at additional time points throughout infection and with a range of infectious organisms, including other entomopathogenic fungus and bacterial pathogens, is required.

### Anapyrexia Mitigates the Reproductive Losses from Infection

Previous work on non‐immunological defences has focused on survival and immunity in infected animals (Thomas & Blanford 2003; Parker *et al*. 2011; De Roode & Lefèvre 2012). To test whether behavioural responses are specific to infected animals, we need to evaluate not just whether infected animals benefit, but also whether they benefit appreciably more from inhabiting preferred temperatures than control animals. Though previous work has argued that behavioural fever and anapyrexia provide survival benefits for infected animals (Müller & Schmid‐Hempel 1993; Adamo 1998; Elliot, Blanford & Thomas 2002), we find that survival benefits are not sufficient to explain why infected animals prefer colder temperatures. While cold‐seeking behaviour indeed enhances the survival of *Metarhizium*‐infected fruit flies, uninfected control flies also receive survival benefit by residing at colder temperatures. The survival benefit of colder temperature does not derive from reduced rates of ageing (in control animals) or physiological decline (in infected animals). Instead, lower temperature greatly reduces the background risk of death, but its influence was relatively similar within each treatment.

At the preferred cooler temperature, infected animals exhibited an increased LRS, but there was no evidence that they benefited more than control animals. Instead, we find that inhabiting cooler temperatures facilitates a shift in life‐history strategy that is specific to infected animals: enhanced late‐age reproduction coming at a cost to early‐age reproduction (supported by a three‐way interaction between temperature, infection and age). We found that infected fruit flies choose a temperature that reduces their fecundity immediately post‐infection (days 2–4 post‐infection), but provides enhanced reproduction at later time intervals (from day 6 onwards) both in terms of the number of eggs laid and the number of fecund days. Although it is important to note that reproductive output is not equivalent to reproductive effort, this finding is consistent with host‐mediated fecundity reduction strategy (Hurd 2001) and runs contrary to the strategy of fecundity compensation (Forbes 1993). As infected flies also achieved greater LRS at anapyrexia‐like temperature compared to those residing at the temperature preferred by control animals, our results highlight evidence of the costs of parasitism (e.g. a temporary reduction in host fecundity post‐infection) can be misleading. The adaptive values of fecundity compensation or reduction are likely to depend on the demography of the population. In particular, fecundity compensation might be maladaptive for declining populations associated with pathogen‐rich environments where late‐age reproduction is likely to contribute more to overall fitness. Whereas a fecundity reduction strategy with relatively higher late‐age reproduction is expected to be adaptive in declining populations such as those experiencing a high parasite burden (Charlesworth 1994; Brommer 2000).

### Anapyrexia is Costly for Fruit Flies

A central concept in ecological immunology is that all host defence traits have costs which are traded off with other host life‐history traits (Sheldon & Verhulst 1996; Schmid‐Hempel 2003). This is an important prediction because in the absence of costs, we would expect defence traits to be constitutively expressed with minimal variation among individuals. We find that the anapyrexia‐like temperature significantly reduced early‐age fecundity and intrinsic rate of increase, *r* in uninfected control flies. These are likely to represent significant fitness costs in growing populations where the intrinsic rate of increase is an appropriate measure of Darwinian fitness and to which early‐age reproduction has a disproportionate contribution (Charlesworth 1994; Brommer 2000). Thus, poikilothermic animals in expanding populations are expected to favour fast development and early‐age reproduction, both of which are positively influenced by ambient temperature (Taylor 1981; Huey *et al*. 1995; Dillon, Cahn & Huey 2007), in order to maximize their intrinsic rate of increase (Huey & Berrigan 2001).

Previous studies using *D. melanogaster* indicate that adult flies have a strong temperature preference at approximately 24–25 °C (Sayeed & Benzer 1996; Dillon *et al*. 2009), we confirm this in uninfected control flies and show that they achieve higher intrinsic rates of increase at 25 °C than those kept at 22 °C. This is consistent with the previous finding that in Drosophila*, r* is maximized at 25 °C (Siddiqui & Barlow 1972; Martin & Huey 2008). In contrast, we found that LRS was not significantly different at the two temperatures in uninfected control treatments. Along with a recent study in nematodes (Anderson *et al*. 2011), our results suggest that at least in some populations of poikilotherms, the temperature preferences of uninfected animals might have evolved to maximize intrinsic rate of increase and not LRS. Together, these results suggest that if we only conduct our experiments under a single thermal condition, which does not represent the highly variable environment experienced by organisms under natural conditions, we may not be able to accurately assess the costs and benefits of immunity. In particular, phenotypes which result from genetic and environmental interactions may be missed (Moret & Schmid‐Hempel 2004; Lazzaro & Little 2009; Paaijmans *et al*. 2013).

### Conclusions

Our findings show that fruit flies alter their temperature preference during an infection. This has implications for the reproductive success of fruit flies and may facilitate a mechanism for poikilotherms to tailor their life‐history strategies in response to infections. We demonstrate the importance of accounting for the thermal environment in studies of host–pathogen interactions and highlight that measuring classic fitness measures, LRS and *r*, within the same experiment can yield novel insights (Huey & Berrigan 2001; Anderson *et al*. 2011). Most importantly, we hope that these results stimulate further experimental work that directly assesses the importance of this mechanism in thermally variable environments including wild populations where poikilotherms are subject to fluctuating environmental temperatures. Recent studies suggest that behavioural thermoregulation will be a key mechanism for poikilothermic animals to buffer the impacts of global climate change (Kearney, Shine & Porter 2009; Gvozdík 2012). Moreover, given the recent global spread of fungal pathogens (Fisher *et al*. 2012), thermoregulatory behaviours are likely to play increasingly important roles in defending against these threats. Wild populations are also regularly under the threat of pathogenic exposure, and this can be variable in both the type of pathogen present and their virulence. We would expect that consequently the degree to which an infection, or more realistically, co‐infections of pathogens affect the fecundity and longevity of an individual is itself subject to great variability. The work we present here is an important step in our understanding of how an animal may mitigate these costs of infection.

## Supporting information


**Appendix S1.** Supplementary methods.

**Fig. S1.** Apparatus for measuring temperature preference of *Drosophila*.
**Fig. S2.** Temperature preference in the Oregon‐R laboratory strain of *Drosophila melanogaster*.
**Fig. S3.** Age‐specific fecundity patterns of *Drosophila* under different temperature and pathogen treatments.
**Fig. S4.** Fitness consequences of cold‐seeking behaviour.
**Fig. S5.** Cooler temperature enhances survival in all pathogen treatments across independent experiments.
**Fig. S6.** Temperature did not influence the relationship between pathogen load and host mortality (tolerance) of flies infected with *Metarhizium*.

**Table S1.** Mixed effects model terms and significance for overall agespecific fecundity models.
**Table S2.** Mixed effects model terms and significance for individual age‐specific fecundity models.
**Table S3.** Analysis of variance terms and significance for the effect of pathogen and temperature on two fitness measures.
**Table S4.** Mixed effects model terms and significance for age‐specific pathogen load.
**Table S5.** Mixed effects model terms and significance for the effect of temperature on the relationship between pathogen load and host mortality (tolerance).
