# Supplementary material for: Cold‐seeking behaviour mitigates reproductive losses from fungal infection in Drosophila
Source: J Anim Ecol. 2015 Oct 16;85(1):178–86. doi: 10.1111/1365-2656.12438 (PMC4879349; doi:10.1111/1365-2656.12438)
Supplement: Supplementary file 1 — Appendix S1. Supplementary methods. [file JANE-85-178-s001.docx]

**Supplementary Methods**

*Statistical analyses*

We analysed temperature preference with chi-squared tests by pooling flies from all replicate trials and dividing them into three approximately equal temperature categories along the temperature gradient: cold (~16-22 °C), medium (~22-27.7 °C) and warm (~27.7-32 °C) (see **Supplementary Figure 1**). For host survival and mortality, we first fitted the Cox proportional hazard regressions with pathogen, temperature and pathogen × temperature using female survival data (models fitted using males gave qualitatively similar results). We then converted the survival data into natural-log transformed mortality rates before fitting the age-specific mortality models [1]. The parameters of the age-specific mortality models were estimated using the minimised sums of squares and maximum likelihood methods in the R package ‘Survomatic’ (version 1.4.0.0). We combined the sexes for model fitting as we did not find any sex differences in mortality patterns. Individual Gompertz models and the more complex logistic models were fitted to each pathogen/temperature treatment combination. The Gompertz model describes the classic pattern where mortality increases exponentially with age and has the hazard function:

*ae^bt^*

where *t* is the age at death, *a* is the morality intercept (background or age-independent mortality), and *b* is the rate of increase in mortality (rate of ageing). The Logistic model modifies Gompertz model by adding the *s* parameter, the rate of deceleration of mortality at older ages (mortality levelling-off) and is described by the hazard function:

*ae^bx^[1 + (a s/b)(e^bx^ – 1)]^-1^*.

Log-likelihood ratio tests were used to assess the significance of the differences between estimated parameter values for all treatments.

We assessed the effect of temperature and infection on two measures of reproductive fitness (LRS and *r*). LRS was measured as the total number of eclosed pupae over the entire lifetime for each female; *r* was estimated for each treatment combination within each block using the number of eclosed pupae produced at each collection interval (n=30). We obtained estimates of *r* by solving numerically the discrete form of the Euler-Lotka equation:

1 *=* Σ *e^-rx^ l_x_m_x_*

where *x* is the age class, *l_x_* is the probability of surviving from age class *x* to age *x+1*, and *m_x_* is the expected number of offspring for a female in age class *x* [2]. We performed separate ANOVAs on LRS and *r* that included pathogen, temperature, and pathogen × temperature. To examine the effects of temperature and infection on age-specific fecundity, we fitted separate linear mixed effects models to eggs and pupae data. The full models included eggs or pupae as the response variable; age, pathogen, temperature and all their interaction terms as fixed effects; Individual females were treated as a random effect. We excluded fecundity data in the first two-day interval (day 2-4 post-inoculation) from analysis of age-specific fecundity to improve the model fit. Repeating the analysis with the first interval yielded similar results. We tested for the 3-way interaction between infection, temperature and age on age-specific reproduction to assess whether temperature preference facilitates life history trade-offs between early and late-age reproduction that is specific to infected animals. We tested for temperature-by-infection interactions with respect to survival, mortality rate, lifetime reproductive success or intrinsic rate of increase to evaluate whether inhabiting the preferred temperature enhances an individual trait in infected animals appreciably more than in control animals. And, we conducted conventional statistical analyses between temperature treatments within each infection treatment to independently assess the influence of temperature on life history.

For host resistance, we assessed the effect of host temperature preference on the growth rates of *M. robertsii* both on artificial media and live hosts. For *in vitro* fungal growth, one-way ANOVA was performed on mean daily growth rates on replicate media plates (mm/day). For the growth rate within live hosts, we performed 1) linear regression, with a quadratic term, on the estimated growth rates of individual replicate cages up to peak CFU counts (n=30; ln(CFU)/day); 2) linear regression on the time taken for each replicate cage population to reach peak CFU counts; and 3) mixed effects model on log-CFU counts over time, with temperature and time as fixed effects and cage as a random effect.

For host tolerance, we used mixed effects models to assess the relationship between pathogen load and host mortality across five temperatures. Significant temperature effect on the slope of the correlation between pathogen load and host mortality (pathogen load × temperature interaction) would indicate changes in levels of host tolerance i.e. the ability of the host to maintain low mortality despite increasing pathogen load. At each time point, CFU counts obtained from the same cage were averaged and log-transformed. Age-specific mortalities were estimated by calculating the proportion of flies dying on the day of live fly sampling and two days preceding it (i.e. within 72 hours of live fly sampling). The full model included natural log-transformed age-specific mortality as response variable; pathogen load (ln(CFU)), temperature, pathogen load × temperature as fixed effects; time (number of days post-inoculation) was added as a covariate to account for non-independence of mortality rate estimates over time. Individual cage was fitted as a random effect. The best fit autoregressive error structure (by AIC) treated the measurements as temporally correlated and was implemented using *corAR1()* function in R package 'nlme' [3].

*Effects of temperature on fungal growth, host resistance and tolerance*

Fungal growth measurements were not measured blindly but the differences observed were very clear and consistent with previously published data on *Metarhizium* growth rates [4]. Fungal colonies were observed to grow in a relatively even and circular manner, but to minimise bias in colony size measurement, two perpendicular measurements across the colony were recorded and the mean value reported.

1. Pletcher, S. D. 1999 Model fitting and hypothesis testing for age-specific mortality data. *J. Evol. Biol.* **12**, 430–439. (doi:10.1046/j.1420-9101.1999.00058.x)

2. Charlesworth, B. 1994 *Evolution in Age-Structured Populations*. 2nd edn. Cambridge: Cambridge University Press.

3. Pollitt, L. C., Reece, S. E., Mideo, N., Nussey, D. H. & Colegrave, N. 2012 The problem of auto-correlation in parasitology. *PLoS Pathog.* **8**, e1002590. (doi:10.1371/journal.ppat.1002590)

4. Ouedraogo, A., Fargues, J., Goettel, M. S. & Lomer, C. J. 1997 Effect of temperature on vegetative growth among isolates of Metarhizium anisopliae and M. flavoviride. *Mycopathologia* **137**, 37–43. (doi:10.1023/A:1006882621776)
