# Supplementary material for: Cold‐seeking behaviour mitigates reproductive losses from fungal infection in Drosophila
Source: J Anim Ecol. 2015 Oct 16;85(1):178–86. doi: 10.1111/1365-2656.12438 (PMC4879349; doi:10.1111/1365-2656.12438)
Supplement: Supplementary file 2 — Fig. S1. Apparatus for measuring temperature preference of Drosophila. Fig. S2. Temperature preference in the Oregon‐R laboratory strain of Drosophila melanogaster. Fig. S3. Age‐specific fecundity patterns of Drosophila under different temperature and pathogen treatments. Fig. S4. Fitness consequences of cold‐seeking behaviour. Fig. S5. Cooler temperature enhances survival in all pathogen treatments across independent experiments. Fig. S6. Temperature did not influence the relationship between pathogen load and host mortality (tolerance) of flies infected with Metarhizium. [file JANE-85-178-s002.pptx]

## Slide 1
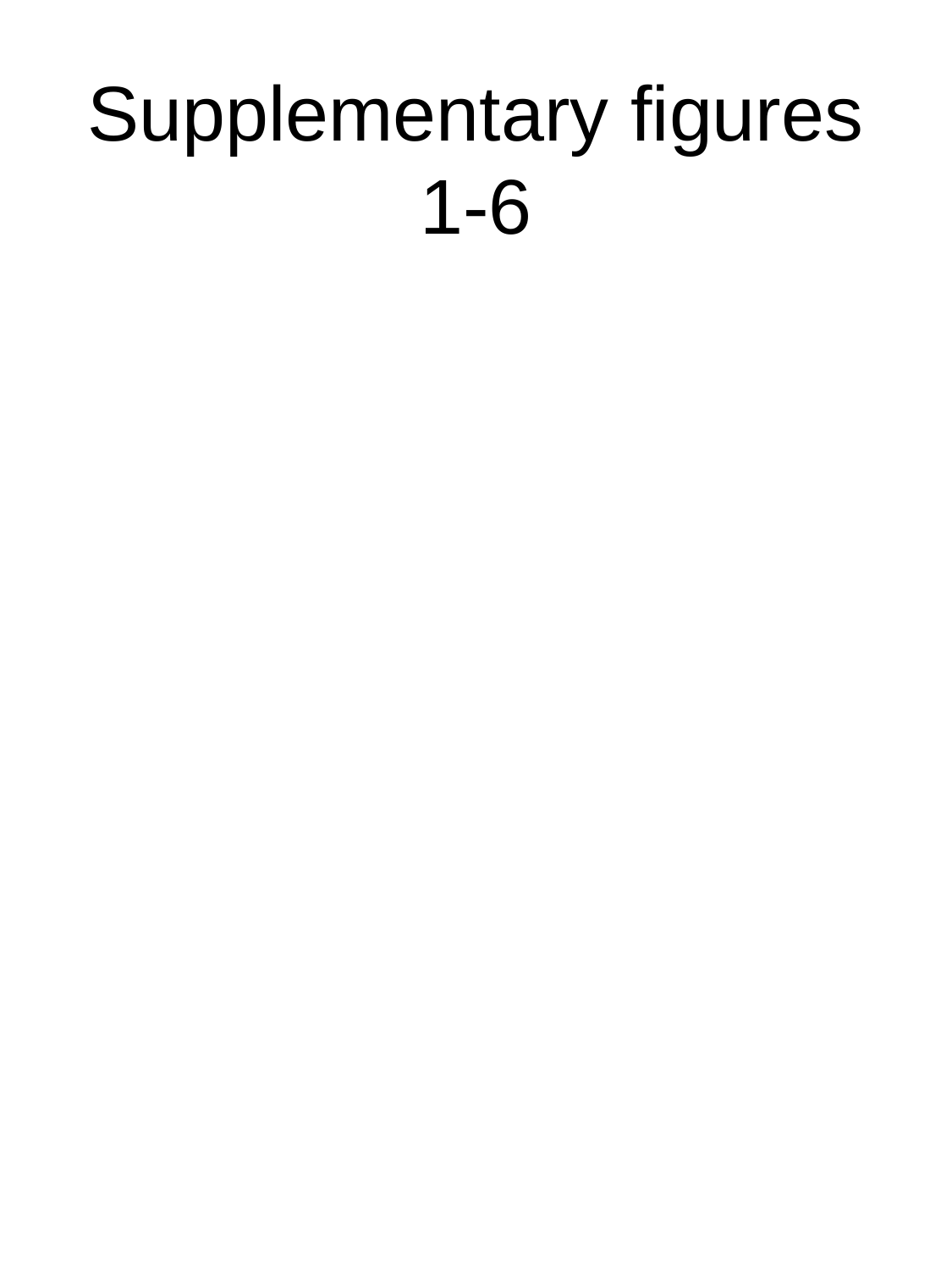

# Supplementary figures 1-6

## Slide 2
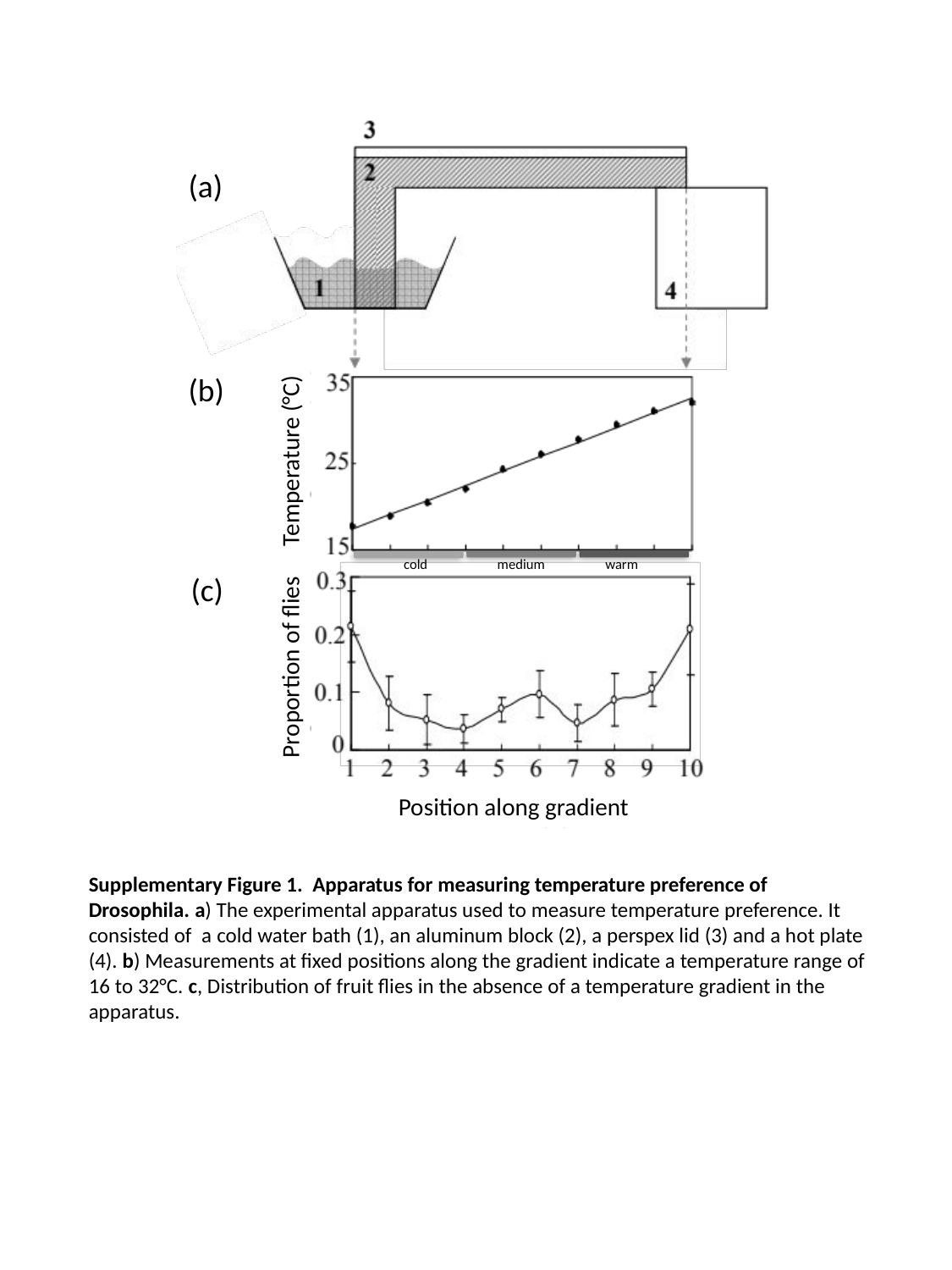

(a)
(b)
Temperature (°C)
 cold medium warm
(c)
Proportion of flies
Position along gradient
Supplementary Figure 1. Apparatus for measuring temperature preference of Drosophila. a) The experimental apparatus used to measure temperature preference. It consisted of a cold water bath (1), an aluminum block (2), a perspex lid (3) and a hot plate (4). b) Measurements at fixed positions along the gradient indicate a temperature range of 16 to 32°C. c, Distribution of fruit flies in the absence of a temperature gradient in the apparatus.

## Slide 3
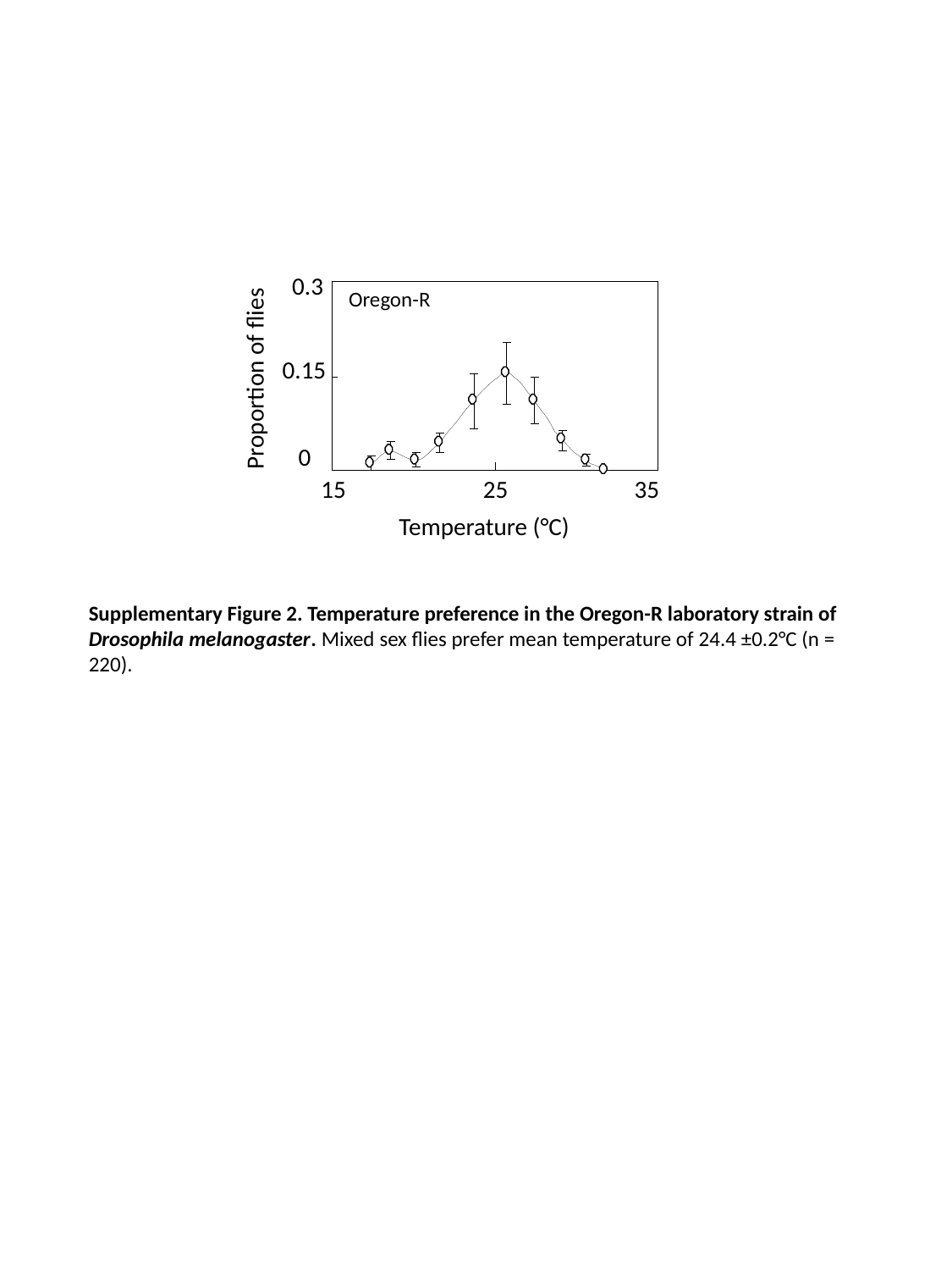

0.3
Oregon-R
Proportion of flies
0.15
0
 15 25 35
Temperature (°C)
Supplementary Figure 2. Temperature preference in the Oregon-R laboratory strain of Drosophila melanogaster. Mixed sex flies prefer mean temperature of 24.4 ±0.2°C (n = 220).

## Slide 4
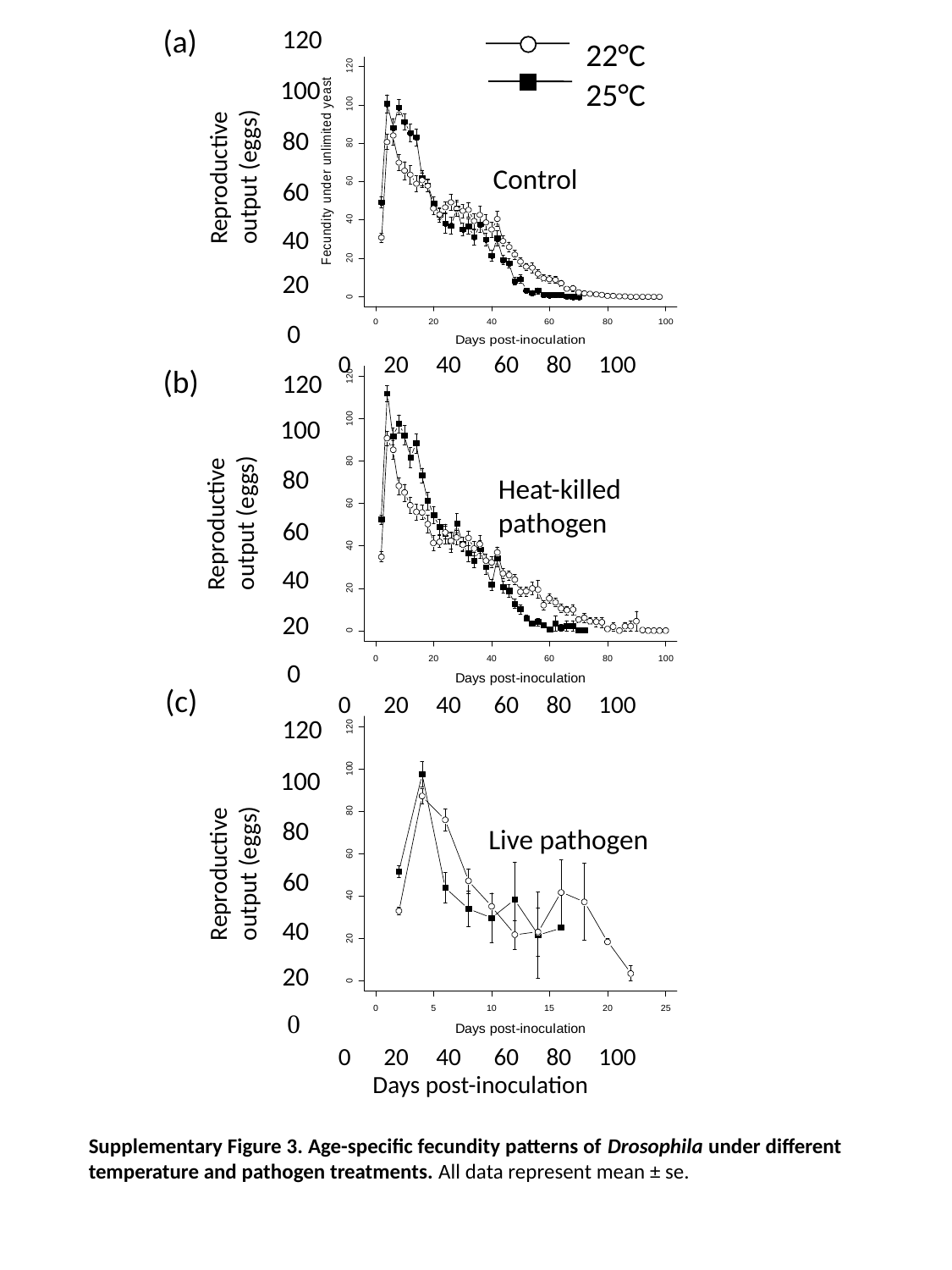

(a)
120
22°C
100
25°C
Reproductive output (eggs)
80
Control
60
40
20
0
0 20 40 60 80 100
(b)
120
100
80
Reproductive output (eggs)
Heat-killed pathogen
60
40
20
0
(c)
0 20 40 60 80 100
120
100
80
Live pathogen
Reproductive output (eggs)
60
40
20
0
0 20 40 60 80 100
Days post-inoculation
Supplementary Figure 3. Age-specific fecundity patterns of Drosophila under different temperature and pathogen treatments. All data represent mean ± se.

## Slide 5
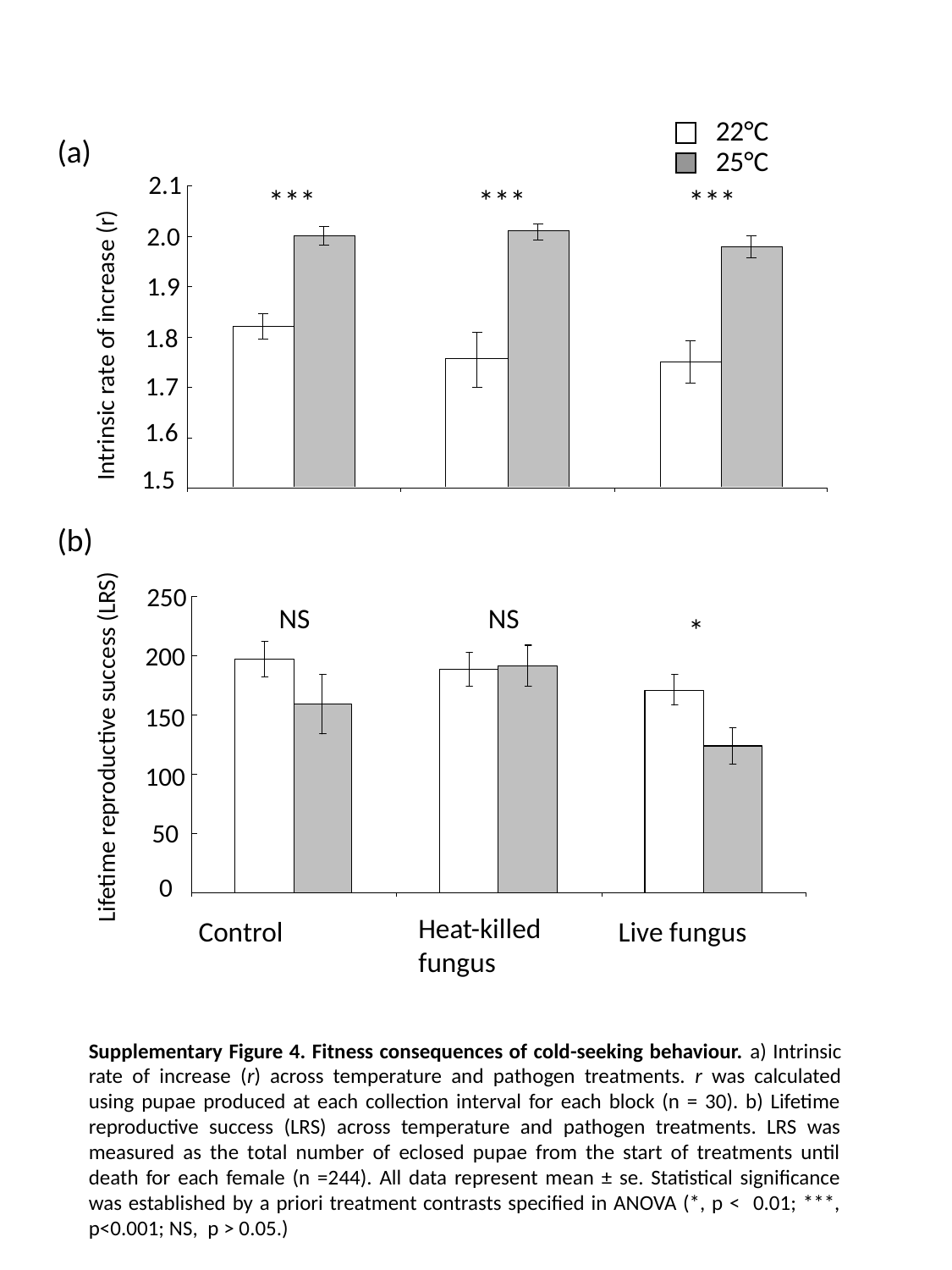

22°C
(a)
25°C
2.1
***
***
***
2.0
1.9
Intrinsic rate of increase (r)
1.8
1.7
1.6
 1.5
(b)
250
NS
NS
*
200
150
Lifetime reproductive success (LRS)
100
50
 0
Heat-killed fungus
Control
Live fungus
Supplementary Figure 4. Fitness consequences of cold-seeking behaviour. a) Intrinsic rate of increase (r) across temperature and pathogen treatments. r was calculated using pupae produced at each collection interval for each block (n = 30). b) Lifetime reproductive success (LRS) across temperature and pathogen treatments. LRS was measured as the total number of eclosed pupae from the start of treatments until death for each female (n =244). All data represent mean ± se. Statistical significance was established by a priori treatment contrasts specified in ANOVA (*, p < 0.01; ***, p<0.001; NS, p > 0.05.)

## Slide 6
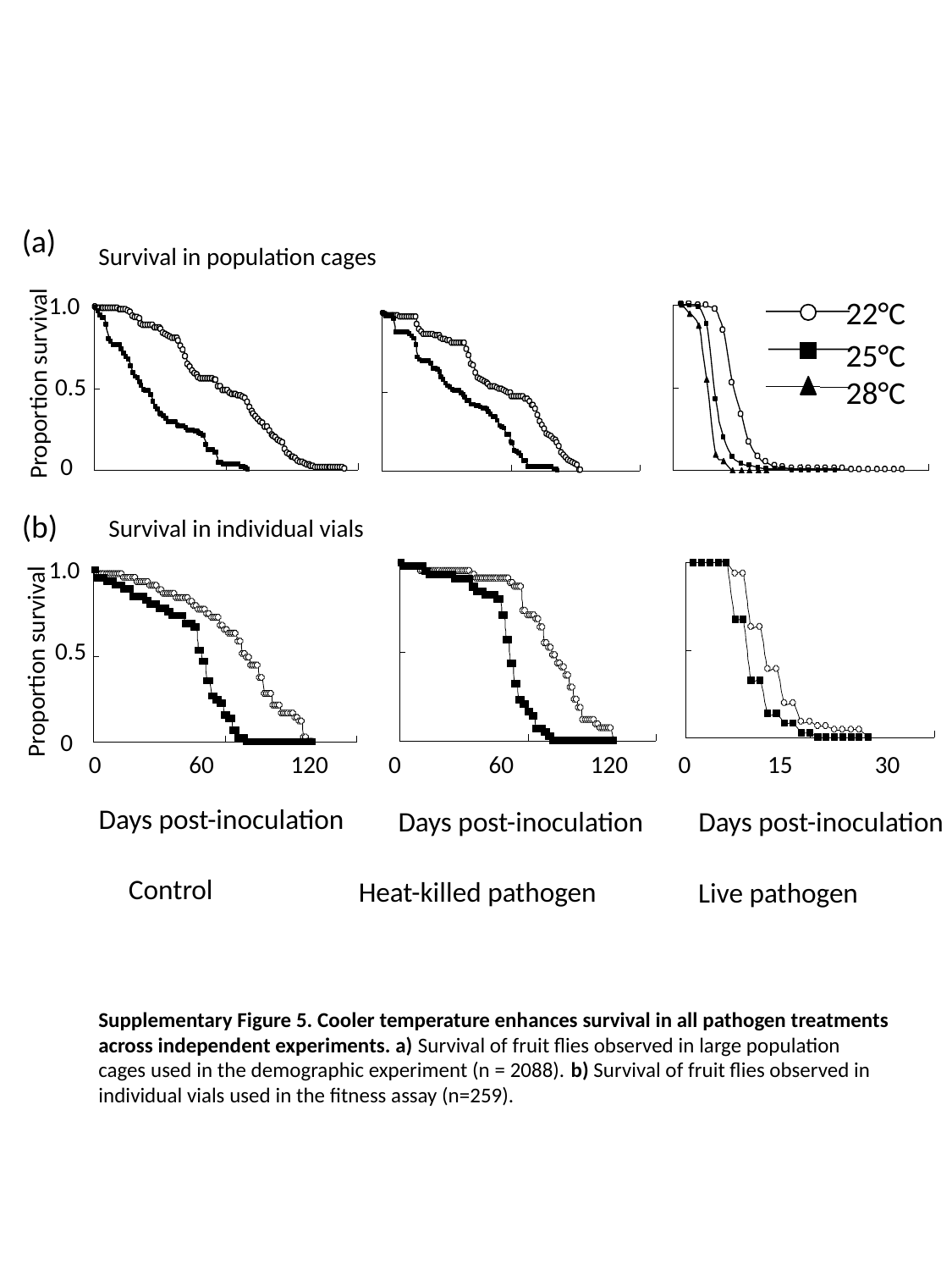

(a)
Survival in population cages
1.0
22°C
Proportion survival
25°C
0.5
28°C
0
(b)
Survival in individual vials
1.0
Proportion survival
0.5
0
0 60 120
0 60 120
0 15 30
Days post-inoculation
Days post-inoculation
Days post-inoculation
Control
Heat-killed pathogen
Live pathogen
Supplementary Figure 5. Cooler temperature enhances survival in all pathogen treatments across independent experiments. a) Survival of fruit flies observed in large population cages used in the demographic experiment (n = 2088). b) Survival of fruit flies observed in individual vials used in the fitness assay (n=259).

## Slide 7
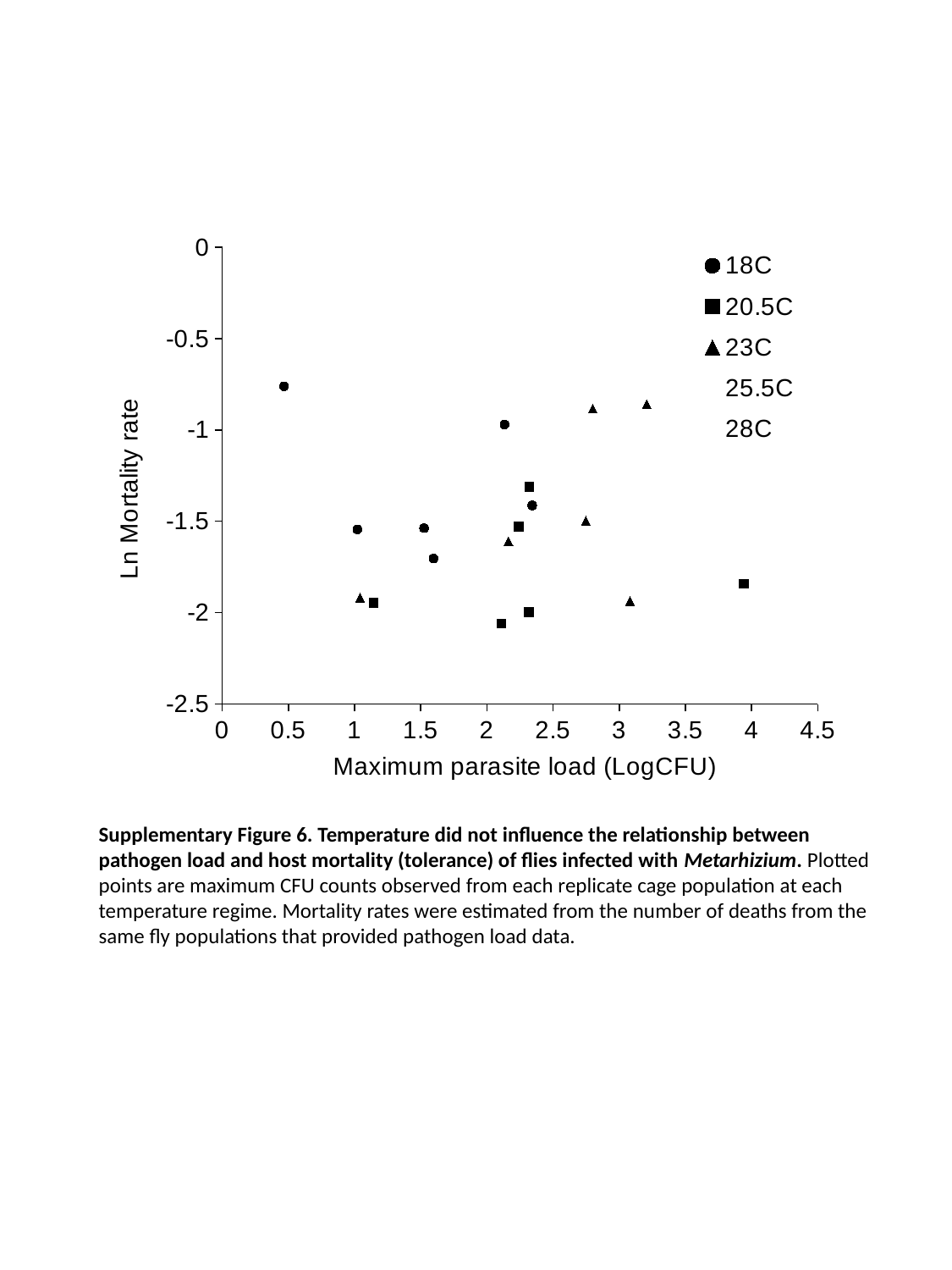

### Chart
| Category | | | | | |
|---|---|---|---|---|---|Supplementary Figure 6. Temperature did not influence the relationship between pathogen load and host mortality (tolerance) of flies infected with Metarhizium. Plotted points are maximum CFU counts observed from each replicate cage population at each temperature regime. Mortality rates were estimated from the number of deaths from the same fly populations that provided pathogen load data.
