# Supplementary material for: Cold‐seeking behaviour mitigates reproductive losses from fungal infection in Drosophila
Source: J Anim Ecol. 2015 Oct 16;85(1):178–86. doi: 10.1111/1365-2656.12438 (PMC4879349; doi:10.1111/1365-2656.12438)
Supplement: Supplementary file 3 — Table S1. Mixed effects model terms and significance for overall agespecific fecundity models. Table S2. Mixed effects model terms and significance for individual age‐specific fecundity models. Table S3. Analysis of variance terms and significance for the effect of pathogen and temperature on two fitness measures. Table S4. Mixed effects model terms and significance for age‐specific pathogen load. Table S5. Mixed effects model terms and significance for the effect of temperature on the relationship between pathogen load and host mortality (tolerance). [file JANE-85-178-s003.docx]

**SI tables**

**Supplementary Table 1.** Mixed effects model terms and significance for overall age-specific fecundity models. Individual females were fitted as random intercepts. We excluded the first egg collection interval post inoculation (day 0-2) in the analysis because accurate model fitting was inhibited by the low levels of fecundity seen in these newly emerged flies.

|  |  |
| --- | --- |
| **Fixed effect** | **Test statistic and p value** |
|  |  |
|  |  |
| Age | F_1,4923_ = 218.3, p < 0.0001 |
| Pathogen | F_2,248_ = 24.0, p < 0.0001 |
| Temperature | F_1,248_ = 27.7, p < 0.0001 |
| Age × Pathogen | F_2,4923_ = 144.6, p < 0.0001 |
| Age × Temperature | F_1,4923_ = 77.3, p < 0.0001 |
| Pathogen × Temperature | F_2,248_ = 0.43, p = 0.65 |
| A × P × T | F_2,4923_ = 15.5, p < 0.0001 |

**Supplementary Table 2.** Mixed effects model terms and significance for individual age-specific fecundity models. Individual pathogen treatments were fitted as separate mixed effects models. Mixed effects model was fitted to the entire data set after excluding the first egg collection interval post inoculation (day 0-2). Individual females are fitted as random intercepts.

| **Fixed effect** | **Test statistic and p value** | | |
| --- | --- | --- | --- |
|  | Control | Heat-killed fungus | Live fungus |
| Age | F_1,2242_ = 171, p < 0.0001 | F_1,2444_ = 264, p < 0.0001 | F_1,237_ = 107, p < 0.0001 |
| Temperature | F_1,83_ = 17.8, p < 0.0001 | F_1,84_ = 33.0, p < 0.0001 | F_1,81_ = 3.4, p = 0.069 |
| Age × Temperature | F_1,2242_ = 52.9 p < 0.0001 | F_1,2444_ = 92.7 p < 0.0001 | F_1,237_ = 9.8, p = 0.002 |

**Supplementary Table 3.** Analysis of variance terms and significance for the effect of pathogen and temperature on two fitness measures. Intrinsic rate of increase was estimated using each treatment combination within each block using the number of eclosed pupae produced at each collection interval (n=30). Lifetime reproductive success was estimated as the total number of eclosed pupae over the entire lifetime for each female. Note that we excluded all females that produced fewer than 5 eclosed pupae prior to the analysis (n=233).

|  |  |  |
| --- | --- | --- |
| **Response variable** | **Effect** | **Test statistic and p value** |
|  |  |  |
| Intrinsic rate of | Pathogen | F_2,24_ = 2.34, p = 0.118 |
| increase (*r*) | Temperature | F_1,24_ = 19.4, p = 0.0002 |
|  | Pathogen × Temperature | F_2,24_ = 1.00, p = 0.384 |
|  |  |  |
| Lifetime reproductive | Pathogen | F_2,227_ = 1.08, p = 0.341 |
| Success (LRS) | Temperature | F_1,227_ = 3.97, p = 0.047 |
|  | Pathogen × Temperature | F_2,227_ = 1.87, p = 0.157 |

**Supplementary Table 4.** Mixed effects model terms and significance for age-specific pathogen load. Model was fitted to log-transformed CFU data set after excluding measurements made on day 5 which only contained estimates for 28°C treatment. Individual cages were fitted as random intercepts.

|  |  |
| --- | --- |
| **Fixed effect** | **Test statistic and p value** |
|  |  |
| Age | F_4,103_ = 3.89, p = 0.007 |
| Temperature | F_1,28_ = 0.17, p = 0.68 |
| Age x Temperature | F_4,103_ = 3.40, p = 0.012 |

**Supplementary Table 5.** Mixed effects model terms and significance for the effect of temperature on the relationship between pathogen load and host mortality (tolerance). Model was fitted to natural-log transformed mortality rates. We used log-transformed CFU data set after excluding measurements made on day 5 which only contained estimates for 28°C treatment. Pathogen load was log10 transformed. Age and temperature were fitted as categorical variables. Individual cages were fitted as random intercepts.

|  |  |
| --- | --- |
| **Fixed effect** | **Test statistic and p value** |
|  |  |
| Pathogen load | F_1,98_ = 4.57, p = 0.035 |
| Temperature | F_4,25_ = 3.31, p = 0.026 |
| Age | F_5,98_ = 107, p < 0.0001 |
| Pathogen load x Temperature | F_4,98_ = 0.96, p = 0.43 |
